# The role of osteocytes-specific molecular mechanism in regulation of mechanotransduction – A systematic review

**DOI:** 10.1016/j.jot.2021.04.005

**Published:** 2021-05-13

**Authors:** Meng Chen Michelle Li, Simon Kwoon Ho Chow, Ronald Man Yeung Wong, Ling Qin, Wing Hoi Cheung

**Affiliations:** a Musculoskeletal Research Laboratory, Department of Orthopaedics and Traumatology, The Chinese University of Hong Kong, Hong Kong, China; b The CUHK-ACC Space Medicine Centre on Health Maintenance of Musculoskeletal System, The Chinese University of Hong Kong Shenzhen Research Institute, Shenzhen, PR China

**Keywords:** Osteoporosis, Osteocyte, Mechanotransduction, Systematic review

## Abstract

**Background:**

Osteocytes, composing over 90% of bone cells, are well known for their mechanosensing abilities. Aged osteocytes with impaired morphology and function are less efficient in mechanotransduction which will disrupt bone turnover leading to osteoporosis. The aim of this systematic review is to delineate the mechanotransduction mechanism at different stages in order to explore potential target for therapeutic drugs.

**Methods:**

A systematic literature search was performed in PubMed and Web of Science. Original animal, cell and clinical studies with available English full-text were included. Information was extracted from the included studies for review.

**Results:**

The 26 studies included in this review provided evidence that mechanical loading are sensed by osteocytes via various sensing proteins and transduced to different signaling molecules which later initiate various biochemical responses. Studies have shown that osteocyte plasma membrane and cytoskeletons are emerging key players in initiating mechanotransduction. Bone regulating genes expressions are altered in response to load sensed by osteocytes, but the genes involved different signaling pathways and the spatiotemporal expression pattern had made mechanotransduction mechanism complicated. Most of the included studies described the important role of osteocytes in pathways that regulate mechanosensing and bone remodeling.

**Conclusions:**

This systematic review provides an up-to-date insight to different steps of mechanotransduction. A better understanding of the mechanotransduction mechanism is beneficial in search of new potential treatment for osteoporotic patients. By delineating the unique morphology of osteocytes and their interconnected signaling network new targets can be discovered for drug development.

**Translational potential of this article:**

This systematic review provides an up-to-date sequential overview and highlights the different osteocyte-related pathways and signaling molecules during mechanotransduction. This allows a better understanding of mechanotransduction for future development of new therapeutic interventions to treat patients with impaired mechanosensitivity.

## 1 Introduction

Ageing population is a major concern worldwide. People generally perform less amount of load bearing exercises during ageing and become more prone to falls and fractures. Less mechanical stimulation on skeletal system for maintenance of its normal function and metabolism will further deteriorate older people's bone health and mobility.

Osteoporosis is a common degenerative disease in aged population [1]. Regular load bearing exercises are crucial for maintenance of skeletal system. Bone can adapt to its mechanical environment with continuous remodeling, which keeps orchestrating bone formation and resorption [2]. Mechanotransduction is a process that transduces the mechanical stimulation from loading to biochemical signals, thus inducing responses of bone cells. Osteoporotic bones are believed to have defective mechanotransduction, as aged cells are less sensitive to mechanical loading and there is a higher proportion of bone forming cells undergoing apoptosis [3,4]. As a result, bone resorption rate is relatively higher in osteoporotic bone and its microstructure will deteriorate, which bones become more brittle [1]. Also, decrease in estrogen receptors in osteoporotic patients is proven to impair the anti-apoptotic effect of loading, thus lowering osteoblasts/osteocytes’ sensitivity to mechanical loading [5,6].

Osteocytes make up 90% of bone cells and are well known for its function as mechano-sensor and transducer. Osteocytes are essential for bone to adapt to mechanical environment, as their morphology and endocrine functions play important roles in transducing mechanical signals to biochemical responses. The cell processes of osteocytes can extend and connect to neighbouring cells fusing the pericellular space around osteocyte cell body and form lacunar canalicular network (LCN). The interconnected cells can communicate via gap junctions and the extended osteocyte network will increase the coverage of signal transmission [7]. Bone surface lining cells, osteocytes and marrow cells are connected by this network which will enhance the load sensitivity and communication among cells [8]. Interstitial fluid can flow freely in the LCN bringing substances in and out of the system [9,10]. Osteocytes in aged bone showed fewer tethering branches and abnormal nucleus which will affect the normal functioning of osteocytes in mechanotransduction [11]. Recent studies have shown that osteocytes can regulate bone remodeling by altering the expression of signaling molecules such as nitric oxide, calcium ions and piezo1 [[12], [13], [14], [15]]. The mechanical signals received by osteocytes will activate the regulation on bone remodeling and respond according to strain level [16]. For example, Piezo1 is important in mechanotransduction as it can modulate calcium influx via Piezo1/2 dependent Ca^2+^ signaling and coordinate bone homeostasis via regulating osteoblast or osteoclast differentiation [15,17,18]. Osteoblasts and osteoclasts are effectors of osteocyte-mediated mechanotransduction, by which the release of sclerostin and receptor activator of nuclear factor κ B- ligand (RANKL) from osteocytes can regulate osteoblastic and osteoclastic activities [19].

There are more studies delineating the mechanism of osteocyte mechanotransduction function in recent years. Mechanical signals can be transduced to biochemical signals by a few pathways. Furthermore, there are various proteins and signaling molecules involved in mechanotransduction and their associations are still poorly understood. This systematic review aimed to outline the sequence of activating different pathways and signaling molecules during osteocyte mechanotransduction. Dysfunction may happen at different stages of mechanotransduction when body homeostasis becomes impaired. The understanding of these mechanisms can facilitate the development of potential therapeutic targets for treating ageing-induced bone disorders.

## 2 Materials and methods

### 2.1 Search strategy

Literature search was performed in PubMed and Web of Science databases (last accessed on 9 Mar 2021). The keywords used in search strategy were “osteocyte∗” AND “mechanotransduct∗“.

### 2.2 Search criteria

The inclusion criteria were: 1) pre-clinical or clinical studies on mechanotransduction function of osteocytes; 2) involving mechanical load stimulation; 3) full-text literature in English; 4) literature published within recent 10 years.

The exclusion criteria were: 1) review paper; 2) conference abstract; 3) non-English; 4) non-osteocyte related; 5) diseases related; 6) unloading under non-physiological situation.

### 2.3 Selection of studies

Study selection was conducted by two independent reviewers. Titles and abstracts were primarily screened to exclude irrelevant studies. Studies were selected based on inclusion and exclusion criteria and disagreements were resolved by discussion and consensus.

### 2.4 Data extraction

The following information was extracted by reviewers: study model used, species of animal or cell type used, form of mechanical stimulation, signaling molecules or pathway involved, transduction mechanism and assessment outcomes.

### 2.5 Data analysis

The studies included in this review adopted various study models and different methodologies. There were also discrepancies in assessment outcomes and statistical methodologies, which meta-analysis was not suitable to be conducted. A qualitative review was performed on the mechanism involved in osteocyte mechanotransduction.

## 3 Results

**Figure 1:**
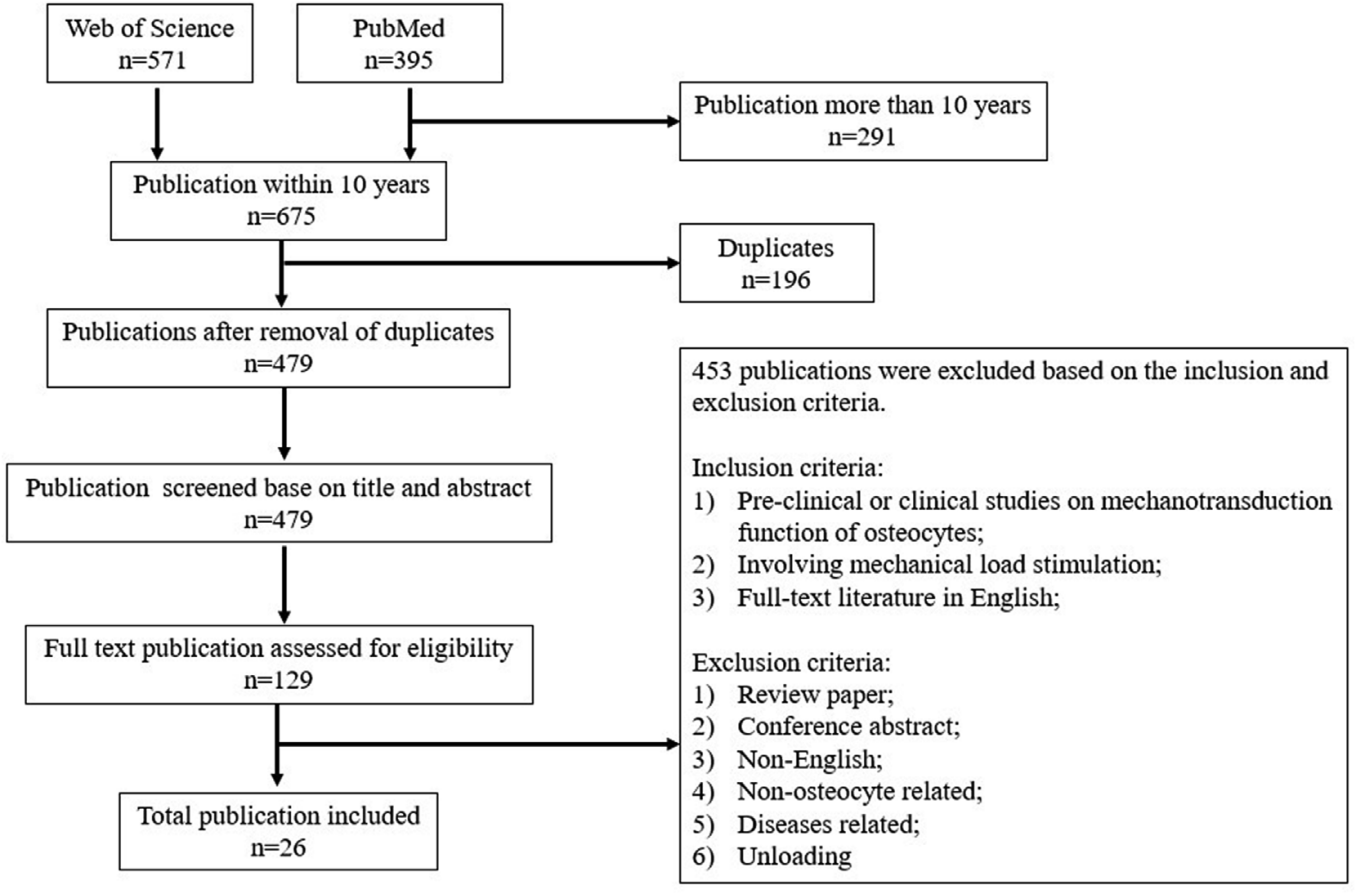
Flow chart for selection process.

A total of 395 and 571 studies were identified from PubMed and Web of Science respectively. Articles that were duplicated and published more than 10 years were removed, leaving 479 studies. After screening all titles and abstracts, 129 studies were identified for further review based on the selection criteria. Upon detailed review of each study in full text, an additional 103 studies were excluded, out of which being conference abstracts and review papers, unloading model used different from normal physiological load, development of bioreactors and imaging technology investigation. Our results included a total of 26 manuscripts for analysis. The flow diagram in Fig. 1 summarizes the selection process.

### 3.1 Characteristics of the papers

The 26 studies were published from 2010 to 2020. Table 1 showed a summary of the included studies. There were 25 pre-clinical studies and 1 clinical study. Seventeen were *in vitro* studies [[20], [21], [22], [23], [24], [25], [26], [27], [28], [29], [30], [31], [32], [33], [34], [35], [36]], 5 were combined *in vitro* and *in vivo* studies [[37], [38], [39], [40], [41]], 3 were *in situ* studies [[42], [43], [44]] and 1 included both clinical and *in vitro* studies [45]. MLO-Y4 murine osteocyte-like cells were used in 18 studies [20,21,[23], [24], [25], [26], [27], [28], [29],[31], [32], [33],[35], [36], [37], [38],^40^,^45^]. Two of the studies used Ocy454 murine mature-osteocytic cell line [22,30], 1 study used IDGSW3 murine intermediate-osteocytic cell line [34], 1 study used isolated primary osteocytes [41] and 1 study used primary murine bone cells composed of osteoblasts and osteocytes [39]. All selected *in vivo* and *in situ* studies used C57 mice model.

**Table 1:** Summary of included studies.

Mechanical stimulation		Magnitude	Model	Species	Reference(s)
Fluid flow shear stress	Oscillating	10 dynes/cm^2^	*In vitro*	MLO-Y4	[21, [23], [24], [25], 27, 28, 31]
				Primary murine bone cell	[39]
		Other than 10 ​dyn/cm^2^, ranging from 2 to 35 ​dyn/cm^2^	*In vitro*	MLO-Y4	[29, 33, 38, 40, 45]
				Ocy454	[22,30]
	Single pulse	310ul/s,	*In vitro*	MLO-Y4,	[26]
		30 ​dyne		Isolated primary osteocyte	[41]
		1 ​V 10 ​Hz	*In situ*	C57 femur	[43]
Stretch		2–5 ​Hz	*In vitro*	MLO-Y4	[23,37]
				IDG-SW3	[34]
Compressive axial load		2–3 ​N, 2 ​Hz, 120 cycles	*In vivo*	C57	[[37], [38], [39]]
		2 ​N, 2 ​Hz, 100 cycles			[40]
		4–8 ​N	*In situ*	C57 tibia	[44]
Physiological load		N/A	*In situ*	C57 femur	[42]
		N/A	Clinical	Human spine and iliac crest	[45]
Drop medium		1–140 ​dyn/cm^2^	*In vitro*	MLO-Y4	[20]
Spectrin disruption		N/A	*In vitro*	MLO-Y4	[35]

### 3.2 Mechanical stimulation

Various mechanical stimulation methods were used in the 26 studies included in this review. Oscillatory fluid flow was applied in 17 *in vitro* studies [[21], [22], [23], [24], [25],[27], [28], [29], [30], [31], [32], [33],^36^,[38], [39], [40],^45^]. Shear stress induced by fluid flow was used to stimulate osteocyte-like cells in the studies. The resulting shear stress applied to the cells ranged from 2 ​dyne/cm^2^ to 35 ​dyne/cm^2^, where 9 out of the 17 studies used 10 ​dyne/cm^2^ as parameter of mechanical stimulation. Two studies applied single fluid pulse to the *in vitro* models [26,41] and 1 study [43] to their *in situ* model, which also created shear stress. Syringe pump could directly apply cyclic hydraulic pressure loading of 0.5 ​Hz, 68 ​kPa to osteocyte cell culture and initiate mechanical response as shown in the study of Liu et al. [46]. Three of the studies [23,34,37] applied stretching force to cells which affected their strain level environment. One study [20] stimulated cells by dropping medium directly above, creating shear stress between 1 and 140 ​dyne/cm^2^. Compressive axial loading was applied to the limbs of experimental animals in 5 *in vivo* and *in situ* studies [[37], [38], [39], [40],^44^]. Two studies [42,45] assessed osteocytes from mice femur, human lumbar spine and iliac crest biopsy samples treated with physiological load. One study [35] induced disruption of cytoskeletal proteins, spectrin and F-actin network, aiming to simulate the activation of signal transduction after mechanical loading. The network connecting microtubules and plasma membrane was disrupted which created strain similar to the tension after contraction of cytoskeleton.

### 3.3 First stage of mechanotransduction: Mechanical sensing proteins

Osteocytes are well known for sensing mechanical signals. Interstitial fluid flow around osteocytes provides the mechanical stimulation to trigger mechanical signal transduction process. Molecules located along the osteocyte dendrites acted as sensors to receive signals from the bone surface, while the outreaching dendrites increased contacting surface to surrounding.

#### 3.3.1 Connexins 43

Osteocytes in LCN extend through a long distance and are interconnected by cell processes. They can communicate with surrounding cells via gap junctions formed by connexin proteins mediated hemichannels and transmit signals. Five of the included studies [20,29,32,37,42] examined expression of Connexins 43 (Cx43) hemichannels under mechanical stimulation. Cabahug-Zuckerman et al. demonstrated that Cx43 uniformly distributed in osteocytes and could function independently without forming cluster with β_3_ integrin [42]. Both Li et al. and Ren et al. showed an increase in Cx43 expression with application of 16 ​dyn/cm^2^ and 12 ​dyn/cm^2^ of fluid flow, respectively, indicating the opening of hemichannels and exchange of molecules in the presence of mechanical load [29,32]. They also suggested that gap junctions were important for mechanical load-induced prostaglandin E2 (PGE2) release to regulate bone remodeling. Burra et al. found that 5.7 ​dyn/cm^2^ of liquid droplet-induced shear stress applied on the osteocytes dendritic side would cause the opening of hemichannels at cell bodies. Signals were amplified and transmitted along the processes and LCN [20]. In contrast, Bivi et al. showed that absence of Cx43 enhanced periosteal bone formation and increased β-catenin. This indicated a low level of Cx43 might be sufficient for bone remodeling [37]. In summary, hemichannels located at plasma membrane can be activated by mechanical stimulation, as they are at the contacting surface of cell and surrounding mechanical environment.

#### 3.3.2 Transient receptor potential vanilloid 4

Transient receptor potential vanilloid 4 (TRPV4) is a calcium ion channel situated mainly at primary cilium. Three studies assessed the role of TRPV4 in osteocyte mechanotransduction [28,30,31]. Moore et al. proposed that fluid flow at 10 ​dyn/cm^2^ induced osteocyte cilium bending and TRPV4 was activated by the binding of extracellular calcium ions to a calcium modulated protein (Calmodulin domain) on TRPV4 [31]. The opening of TRPV4 allowed Ca^2+^ ions to enter osteocyte cilium. Lyons et al. also demonstrated TRPV4 activation when 4 ​dyn/cm^2^ of fluid flow stimulated cytoskeletons that were attached to TRPV4, causing the influx of Ca^2+^ ions into osteocytes [30]. Furthermore, Lee et al. examined the loss of TRPV4 in osteocyte-like cells which resulted in fewer responsive cells that showed Ca^2+^ oscillation after mechanical stimulation [28]. By summarizing the 5 studies, TRPV4 channel can be opened physically by fluid flow induced movement of cytoskeletons. The extensive osteocyte process network with more dense cytoskeletons can facilitate osteocyte function in mechanotransduction.

#### 3.3.3 Piezo1

Piezo1 is a mechanosensitive ion channel protein which allows transduction of signal upon mechanical stimulation [47]. Sasaki et al. demonstrated that Piezo1-Akt pathway modulated 5 ​Hz cyclic stretch-induced decrease in sclerostin expression in osteocytic cells [34]. The knockout of Piezo1 exerted an inhibitory effect on intracellular Ca^2+^ oscillation and load-induced suppression of sclerostin. This study also showed that Piezo1 in osteocytes induced calcium influx and phosphorylation of Akt which downregulated sclerostin expression when exposed to mechanical load. Piezo1 acted as a sensor and mediated the activation of Akt pathway in osteocytes in order to regulate bone homeostasis.

#### 3.3.4 Sphingosine-1-phosphate

Sphingosine-1-phosphate (S1P) is suggested to be a lipid mediator relating to all signaling pathways involved in osteocyte mechanotransduction. As S1P involves in cytoskeleton organisation and cell adherence, the change in mechanical environment will affect the expression of S1P and further influence bone metabolism [48]. Zhang et al. showed that S1P played a role in flow-induced increase in release of intracellular Ca^2+^ ions [36]. By inhibiting S1P function, fluid flow induced increase in PGE2 and COX-2 expression were suppressed and the reduction of receptor activator of nuclear factor κ B- ligand/osteoprotegerin (RANKL/OPG) ratio was also diminished. Dobrosak et al. demonstrated that fluid flow at 2 ​dyn/cm^2^ stimulated an increase of intracellular S1P in Ocy454 ​cells [22]. Their results also showed that degradation and mobility of S1P were downregulated upon mechanical stimulation, providing more stable S1P release which facilitated osteoblast survival and normal functioning. S1P plays an important role in osteocyte mechanotransduction, as it is capable of coordinating bone homeostasis pathways.

#### 3.3.5 Integrins and kinases

Integrin α_v_β_3,_ Src and Pyk2 kinases are located along the extension parts of osteocytes. They are involved in regulating cell attachment and cytoskeleton structure. Cabahug-Zuckerman et al. showed that α_v_β_3_ integrin cluster could amplify membrane strain via focal adhesion of cell process to canalicular wall [42]. Haugh et al. reported that inhibition of integrin α_v_β_3_ on osteocyte processes diminished flow-induced increase in bone-forming gene expression. Ca^2+^ signaling was also disrupted when α_v_β_3_ was blocked [24]. Hum et al. demonstrated that Src and Pyk2 kinases formed complex and acted as ‘off switch’ that suppressed bone response to mechanical load [25]. α_v_β_3_, Src and Pyk2 relayed mechanical stimulation sensed by cytoskeleton to biochemical response of osteocytes.

#### 3.3.6 Cell cytoskeleton

Spectrin network is responsible for maintaining the stiffness of cells and their mechanical properties. The cell cytoskeleton structure will deform under mechanical stimulation. Wu et al. suggested that spectrin network acted as a mediator of mechanotransduction in osteocytes [35]. They reported that disruption of the spectrin network increased intracellular Ca^2+^ signal and cell–cell connections. These were caused by cell softening that facilitated the mobility of Ca^2+^ ion channels. Liu et al. showed that the change in morphology of loaded osteocyte microtubules was modulated by increase in intracellular calcium ions and further facilitate osteocytes’ response to loading [46]. The distortion of cell structure could activate membrane-associated receptors and initiate signal transduction.

#### 3.3.7 Pericellular matrix

Osteocytes produce pericellular matrix to be laid within the LCN. Mechanical loading induced fluid flow in LCN will lead to deformation of cell membrane and increased shear stress. The drag force together with accumulated shear stress can amplify the strain in LCN under loading [50,51]. This matrix production determines the repair rate of mechanically stimulated plasma membrane disruption (PMD). ATP released from disruption repair process affect the initiation of mechanotransduction. Hagan et al. reported that fluid flow shear stress of 30 ​dyn/cm^2^ lead to PMD in osteocytes and aged osteocytes were less effective in mechanotransduction. Aged osteocytes had faster PMD repair rate that limited time was allowed for ATP production from repair. Downstream mechanotransduction was impaired as less ATP was available for initiating calcium signaling [41]. They also reported that calcium signaling was initiated by the membrane disruption. Pericellular matrix is the frontline contact with signals from environment and plays a role in initiating mechanotransduction.

Apart from the sensing proteins mentioned above, there are many molecules involved in sensing mechanical loading. Table 2 summarizes the molecules located at osteocyte processes and cilium that are activated by interstitial fluid flow. These proteins contribute to mechanosensation function of osteocytes and allow mechanical signals to reach other targets.

**Table 2:** Summary of other sensing proteins.

Molecules	Reference(s)	Findings
Purinergic type 2 (P2X) receptors and Pannexin1 (Panx1)	[26,42]	•P2X7 receptor highly expressed in osteocyte processes •Form complex with stretch activated channel protein Panx1 •Loading activated Panx1, leading to ATP release and P2X7 receptor activation which resulted in Ca^2+^ influx to osteocytes
Calcium channel CaV3	[42]	•Co-localized with integrin complex at focal adhesion site •Integrin-CaV3 cluster created stress concentration •Loading induced Ca^2+^ mobilization, release of ATP and PGE2
Adenylyl cyclase 6 (AC6)	[27,31,39]	•Highly expressed in osteocyte primary cilium •Catalyze conversion of ATP to cyclic adenosine monophosphate (cAMP) •Mediate fluid flow induced decrease in cAMP •Mediate fluid flow induced increase in cyclooxygenase-2 (COX-2) •Inhibition of AC6 impaired bone formation
Zinc finger protein	[45,49]	•Zinc finger protein of cerebellum transcription factor (Zic1) •Interact with Gli1 and Gli3 at osteocyte cilium to regulate bone development via hedgehog pathway •Inhibition of Zic1 abolished the load-induced increase in T cell factor/lymphoid enhanced factor (TCF/LEF) transcription factor of Wnt pathway •Osteocyte cilia movement activated Wnt/TCF1 pathway

In summary, load-induced osteocytes plasma membrane disruption is one of the factors that initiate mechanotransduction [41]. Cytoskeleton deformation upon mechanical stimulation can lead to distorted cell shape and plasma membrane will be disrupted [52]. Strain amplification in LCN induces opening of stretch-activated ion channels as drag force created between cell membrane and canalicular wall deform the channel proteins [50]. Integrin-based adhesion of cells to extracellular matrix allows force transmission which can also trigger signal transduction as mechanosensing-integrins are stretched [53,54]. Transmembrane channels are forced to open by deformation of cytoskeleton, allowing exchange of molecules [55]. Mechanical signals are transduced through increase in expression of channel proteins such as Cx43, TRPV4, CaV3 and Panx1 [29,31,42]. The activation of S1P and P2X7 receptors located along the membrane contribute to the influx of Ca^2+^ when osteocytes are exposed to mechanical load [36,42]. Gli1, Gli3 and AC6 are some of the mediators at primary cilia regulating the activation of Wnt pathway and the expression of COX-2 and cAMP [27,39,45]. Proteins such as α_v_β_3_, Src and Pyk2 are responsible for cytoskeleton attachment to canalicular wall will be mediating the biochemical response from mechanical loading [8,56]. They are highly expressed at focal adhesion sites where fluid flow-induced stress concentrated. These integrin and kinases mediate the increase in PGE2 expression and Ca^2+^ signaling and contribute to mechanosensation under mechanical stimulation [24,25].

There are various signaling molecules mediating osteocyte mechanotransduction but they can be contradicting. Few studies stated that mechanical loading stimulated Cx43 hemichannel expression in osteocytes, thus enhancing bone formation [29,32]. The opened hemichannels at gap junctions facilitate the communication between osteocytes and other neighbouring cells, as there are more passages for the transmission of signals. However, Bivi et al. reported an inhibition of Cx43 would enhance periosteal bone formation [37]. They found that the presence of Cx43 reduced β-catenin level in osteocytes which impaired load-induced bone formation. Cx43 would bind to β-catenin and inhibit Wnt signaling pathway, as translocation of β-catenin was prevented [57]. It was suggested that minimal level of Cx43 was sufficient for signaling molecules transmission, as there were others channel proteins at gap junctions [37].

### 3.4 Second stage of mechanotransduction: Intermediate signal transmission

#### 3.4.1 Calcium ions

Calcium ions play a very important role in osteocyte mechanotransduction. Application of mechanical load initiates rapid response of intracellular calcium ions influx [46,58,59]. These ions act as signals to be delivered to neighbouring cells through ion channels. Ten of the included studies [26,28,30,[34], [35], [36],^38^,^40^,^43^,^44^] had assessed Ca^2+^ ions oscillation when osteocytes were stimulated by mechanical loading. Jing et al. showed the depletion of endoplasmic reticulum Ca^2+^ store caused reduction of calcium oscillation in the osteocytes exposed to mechanical load and number of responsive cells also decreased [44]. Zhang et al. showed that calcium ions oscillation was the early signal response after application of oscillating fluid flow [36]. Four studies [28,30,40,43] showed that fluid flow of 10 ​dyn/cm^2^ induced shear stress, leading to increased intracellular Ca^2+^ ions concentration. Magnitude of Ca spike and percentage of responsive cells were also increased with the application of mechanical stimulation. Ca^2+^ ions acted as signal transmitters to mediate responses further down the mechanotransduction process.

There are few signaling pathways mediating osteocyte mechanotransduction including Akt pathway, Wnt pathway and ERK pathway. β-catenin plays a key role in different pathways. Santos et al. reported that inhibition of focal adhesion kinase (FAK) attenuated fluid flow-induced activation of Wnt/β-catenin signaling pathway. Fluid shear stress of 0.7 ​Pa induced translocation of β-catenin via the Akt pathway, which depended on phosphatidyl inositol 3 kinase (PI3K) activation by binding to FAK. Mechanical signals received at focal adhesion sites led to stabilisation of β-catenin through PI3K/Akt pathway and therefore activated Wnt signaling pathway that modulated bone adaptation to loading [33].

#### 3.4.2 ERK signaling pathway

Extracellular signal regulated kinase (ERK) phosphorylation facilitates proliferation and survival of osteocytes. Both De Castro et al. and Gortazar et al. demonstrated that fluid flow shear stress at 10 ​dyn/cm^2^ induced ERK phosphorylation and imposed an anti-apoptotic effect on osteocytes [21,23]. This pathway was mediated by vascular endothelial growth factor receptor 2 (VEGFR2) and phosphorylation of VEGFR2 was found to be associated with β-catenin translocation, which required the presence of caveolin-1 [21]. Gortazar et al. showed that there was crosstalk between caveolin-1/ERK signaling pathway and Wnt/β catenin pathway. The application of fluid flow induced GSK3β phosphorylation and inhibited degradation of β-catenin, allowing translocation and accumulation of β-catenin [23]. Mechanical load-induced accumulation of β-catenin and activation of ERK were inter-dependent, while inhibition of caveolin-1 abolished both responses.

#### 3.4.3 Nitric oxide

Nitric oxide (NO) is involved in the stabilisation of β-catenin which enables its translocation to nucleus for the activation of Wnt pathway. Santos et al. and Wu et al. showed that fluid flow shear stress at 0.7 ​Pa and disruption of spectrin cytoskeleton network induced NO production [33,35]. NO synthases increased after mechanical stimulation and transferred to cytoplasm where NO synthesis took place. NO contributed to activation of β-catenin mediated Wnt signaling pathway in mechanotransduction process which could regulate bone formation at a later stage.

#### 3.4.4 Adenosine Triphosphate (ATP)

ATP is capable to mediate mechanotransduction in osteocytes. It can be converted to cAMP which is a regulator of bone remodeling. Kringelbach et al. demonstrated that fluid flow of 310 ​μl/s induced acute release of ATP from osteocytes, which was mediated by vesicular exocytosis [26]. Corry et al. reported that ATP increased flow-induced Ca^2+^ ions transients in osteocytes and Stat 3 regulated ATP production from mitochondria [38]. ATP acted as a mediator which induced biochemical responses to regulate bone homeostasis.

#### 3.4.5 Extracellular vesicles

Extracellular vesicles are packed with signaling molecules such as OPG, RANKL and sclerostin. Morrell et al. demonstrated that both fluid shear stress at 35 ​dyn/cm^2^ on *in vitro* model and compression fore of 12 ​N on *in situ* model induced increase in vesicles production form osteocytes [40]. This showed that osteocytes could directly regulate bone adaptation to mechanical load by releasing bone regulating genes in vesicles.

During second stage of mechanotransduction, signaling molecules such as Ca ^2+^ ions and NO are transported via the hemichannels into the cytoplasm. Calcium oscillation is found to be one of the immediate responses to mechanical stimulation [36,60]. Ca^2+^ ions commonly act as signal transmitters that will be delivered to various cell junctions, mediating cell–cell communication. Osteocytes receiving the signals can directly suppress the expression of sclerostin which is an inhibitor of Wnt/β catenin signaling pathway [61]. Moreover, mechanical stimulation can induce an increase of phosphorylated Akt, translocation of β-catenin and activation of Wnt signaling pathway, demonstrating the crosstalk between Wnt pathway and Akt pathway [62]. β-catenin plays an important role in the transduction of mechanical signals and activation of Wnt signaling mainly depends on β-catenin availability. Osteocytes regulate load-induced bone remodeling by mediating mechanotransduction and modulate biochemical responses in osteoblasts and osteoclasts. For example, NO is released from osteocytes via the channel proteins and inhibits osteoclast activities [63]. However, multiple pathways are activated during mechanotransduction and some of them are interdependent. The exact sequence of these events is not yet fully understood and need further investigation.

On the other side, communication between osteocytes and osteoblasts/osteoclasts is not fully elucidated but it is known to be mainly assisted by signaling molecules released from osteocytes. Osteoblasts are responsible for bone formation and β-catenin is an important component in the activation of bone forming genes [64]. Osteocytes experiencing mechanical stimulation suppress the production of sclerostin which allows translocation of β-catenin and activates Wnt pathway in osteoblasts [65,66]. However, at the same time Ca^2+^ sent from osteocytes or extracellular matrix can induce bone growth, showing alternative pathway for enhancing bone formation after mechanical stimulation. Ca^2+^ ions are well known for its role of signal transmitter and also involved in osteocyte regulation of osteoblastic activities apart from sclerostin. A similar situation is also experienced by osteoclasts. RANKL released from osteocytes will regulate osteoclastogenesis, which the mechanism is yet to be fully understood [67]. Ca^2+^ signals arriving osteoclasts lead to acid secretion which facilitates bone resorption [68]. It is a complicated process that multiple events undergo simultaneously to affect each other. The pathways involved in osteocyte mechanotransduction are interdependent and mediated by many signaling molecules, thus causing difficulty to delineate the mechanism. With previous studies on mechanotransduction to clarify the backbone of its process, there are still missing gaps on how mechanical signal transduction happens in precise sequence. There is no solid evidence to prove whether the signal transduction mechanisms happen simultaneously or happen one after the other. Also, the mechanotransduction pathways are interdependent and sometimes involve feedback mechanism. This complicated signal transduction process needs further delineation to reveal the relationships between osteocytes, signaling molecules and the mechanisms behind.

### 3.5 Last stage of mechanotransduction: Effector response

#### 3.5.1 COX-2 and PGE2

COX-2 mediates fluid flow-induced production of PGE2, leading to upregulated bone formation [24]. Eight studies [24,25,27,28,31,36,37,39] reported an increase in COX-2 expression after application of fluid flow shear stress or stretch force on osteocytes. The expression of PGE2 after mechanical load induction were investigated in 6 of the included studies [20,22,24,29,32,36] and showed an increase in PGE2 level. Dobrosak et al. suggested that mechanically induced increase in intracellular calcium would stimulate osteoblastic and osteoclastic activities and caused the increase in PGE2 production and expression [22].

#### 3.5.2 Sclerostin

Osteocytes can regulate bone remodeling by adjusting production of bone formation inhibitor, sclerostin. Xu et al. showed that OCY454 osteocytes response to oscillatory fluid flow by lowering sclerostin expression level and had established intercellular communication to osteoblasts [59]. Sclerostin is an antagonist of Wnt signaling pathway, which suppresses osteoblast differentiation. Five studies [22,29,30,34,40] reported a reduction in sclerostin level with the application of mechanical load. With reduced expression of sclerostin after exposure to fluid shear stress, bone formation was enhanced [29]. This adjusted balance between osteoblastic and osteoclastic activities favoured the gain in bone mass.

#### 3.5.3 RANKL and OPG

RANKL facilitates osteoclastic activities and OPG acts as a decoy receptor for RANKL to inhibit osteoclastogenesis. Osteocytes were found to have a decreased RANKL expression after vibration, which indicated an anti-resorptive effect imposed by osteocytes via altering RANKL/OPG ratio [69,70]. The RANKL-to-OPG ratio indicates the net bone formation or resorption in bone turnover. Three studies [29,32,36] demonstrated that fluid flow shear stress regulated bone remodeling by affecting expression levels of RANKL and OPG. Li et al. reported that the RANKL mRNA expression of MLO-Y4 cells was increased after the induction of 16 ​dyn/cm^2^ fluid shear stress but declined gradually. OPG mRNA expression increased steadily after application of shear stress [29]. Both Ren et al. and Zhang et al. showed a decrease in RANKL/OPG ratio in MLO-Y4 cells after exposure to fluid flow-induced shear stress of 12 and 10 ​dyn/cm^2^ respectively [32,36]. This indicated that bone formation increased after application of mechanical stimulation.

#### 3.5.4 Wnt/β catenin pathway

Wnt signaling pathway plays a key role in mechanotransduction. Osteocytes transduce mechanical stimulation to biochemical response by regulating β-catenin level, thereby releasing signaling molecules to other bone cells [33]. Hu et al. demonstrated that blocking Wnt/β catenin signaling pathway impaired flow-induced Ca^2+^ oscillation and lowered the percentage of responsive osteocytes [43]. This study suggested the possibility of Wnt/β catenin pathway and Ca^2+^ influx crosstalk in osteocytes and they may be interdependent.

#### 3.5.5 Cyclic Adenosine Monophosphate (cAMP)

cAMP inhibitory effect on osteogenesis was shown by increase in COX-2 expression after blocking cAMP production. Two studies reported that flow-induced reduction in cAMP level after exposing to 2 ​min of 10 ​dyn/cm^2^ fluid flow but increased after 30 ​min of flow [27,31]. The transient change in cAMP expression acted as feedback mechanism and it was suggested to restore cAMP level after prolonged flow.

In summary, the expression of bone remodeling genes is altered during the last stage of mechanotranduction, resulting in various biochemical responses. The suppression of Wnt pathway inhibitor sclerostin and the activation of Wnt signaling pathway favour bone formation. Increase in bone formation is mediated by the increase in PGE2 and COX-2 expression and decrease in RANKL/OPG ratio [24,29,32,36,43]. The altered gene expressions modulate osteoblastic and osteoclastic activities during bone adaptive remodeling. However, studies showed that there are conflicting situations in some of the load-induced responses that require further elucidation. The level of cAMP drops immediately when osteocytes are exposed to mechanical stimulation. Kwon et al. reported that cAMP level in osteocytes would increase after exposing to 30 ​min of fluid flow, which was suggested to be a compensatory response [27]. The initial decrease in cAMP can be explained by the rapid Ca^2+^ influx to osteocytes which impair AC6 activity and limit the conversion of ATP to cAMP [71]. The drop in cAMP may be already sufficient to reduce its inhibitory effect on osteogenesis. Also, mechanical load induces upregulation of PGE2 which mediates anti-apoptotic effect and activation of β-catenin signaling pathway [72]. However, Kaji et al. and Amano et al. showed that PGE2 enhanced osteoclastogenesis, which induced bone resorption and osteoclastic activities may initiate further signal transmission to other cells [73,74]. PGE2 functions are mediated by few signaling pathways and PGE2 is able to regulate bone resorption and bone formation depending on cell type and environment.

## 4 Discussion

This review summarizes different signaling molecules and pathways involved in osteocyte mechanotransduction. The process of transducing mechanical signals to biochemical responses were also illustrated in this review. Osteocytes with extended cell processes are well known for mechanosensation and transduction of mechanical signals to neighbouring bone cells which regulate bone turnover. Transmission of downstream signaling molecules through gap junctions can reach osteoblasts or osteoclasts and regulate their activities in bone remodeling. Osteocytes are the most abundant cells in bone and the network can spread across a great area. Osteocytes in the widespread network can sense mechanical stimulation on bone surface and transduce the mechanical signals to other distant cells. Osteocyte processes increase cell surface area and the primary cilia will also increase accordingly, which elevate osteocytes’ sensitivity to mechanical stimulation [75]. Studies showed osteocytes play an essential role in mechanotransduction to link up different mechanisms and regulate bone remodeling.

**Figure 2:**
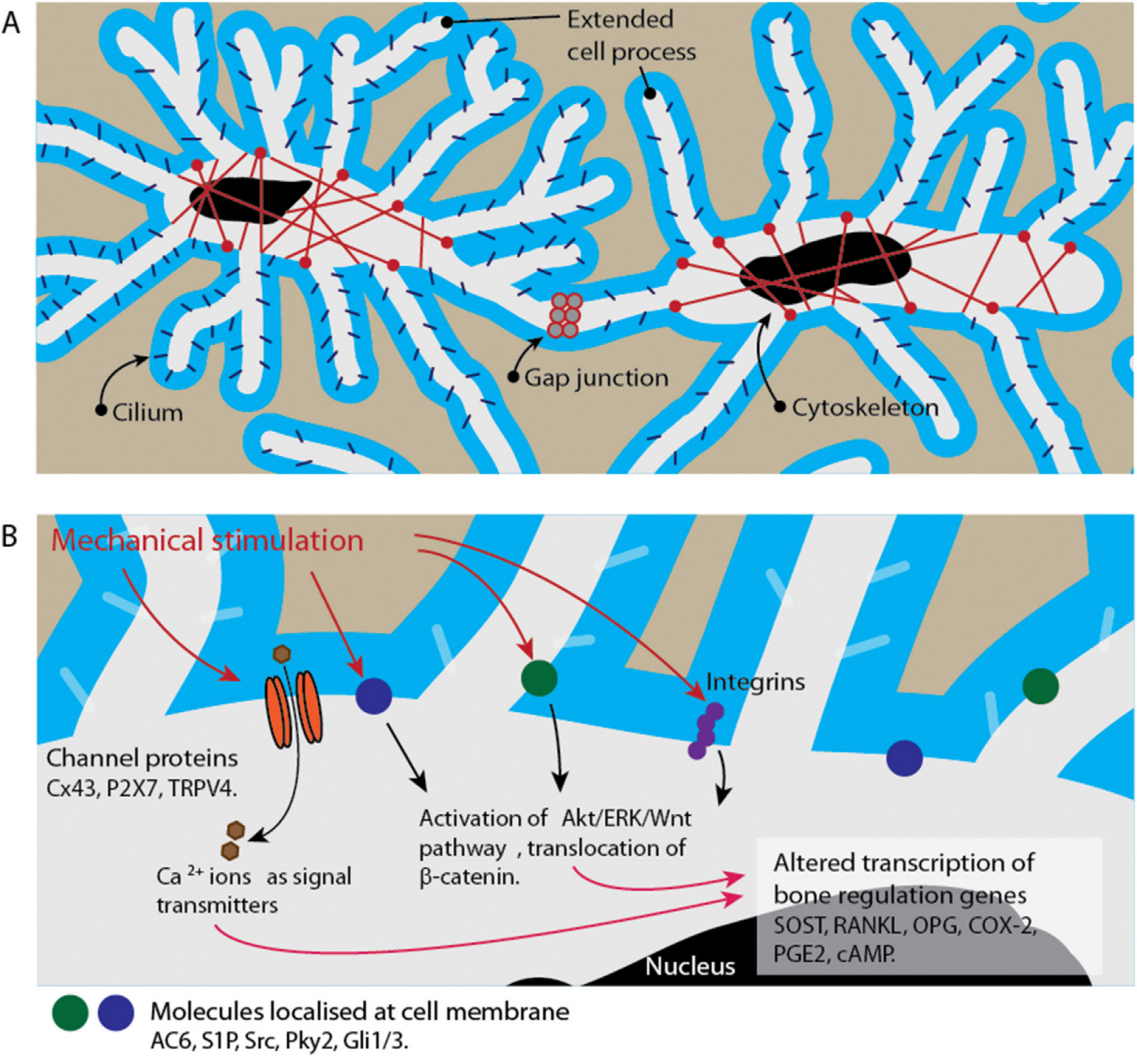
Overview of the role of osteocyte-specific molecules in mechanotransduction. Osteocytes are interconnected to form LCN that facilitates mechanotransduction. Pericellular matrix contributes to sensation of load-induced interstitial fluid flow. Cell processes and cilium are connected to cytoskeleton and the deformation of structure can initiate signals transmission. Gap junctions between cells are responsible for direct exchange of signaling molecules. Proteins located at the extension parts and membrane can mediate mechanotransduction through a few pathways. The expression of bone remodeling genes will be altered when adapting to the changes in mechanical environment.

Mechanotransduction can be categorized into 3 different stages. Fig. 2 shows the sequence of mechanotransduction process. Osteocytes form LCN and mechanical load induces interstitial fluid to flow through LCN in bone matrix [50,51,76,77]. The flow will change the strain level which leads to deformation of osteocytes LCN and the cytoskeletons extend along cell processes [50,51]. Moreover, few studies showed that primary cilia on osteocytes processes were sensitive to strain environment and cytoskeleton could be triggered by cilia movement [28,39]. These mechanical signals are transmitted along the osteocytes processes and passed to surrounding cells via gap junctions and ion channels [32,78].

The bending of cilia and contraction of cytoskeleton happen simultaneously upon mechanical loading, leading to direct opening of transmembrane channels and ATP mediated activation of channel receptors respectively. Extracellular ATP are required to initiate the contraction of cytoskeleton. ATP will be further produced and activate P2X receptor gated channel, allowing Ca^2+^ influx to osteocytes [40,44]. Though P2X receptors are direct effector of mechanical stimulation, the activated gated channel can facilitate signal transmission by allowing Ca^2+^ ions to be transported to other targeting effectors. Both pathways eventually mediate Ca^2+^ oscillation in osteocytes exposed to mechanical stimulation.

Considering the 26 included studies in this review, osteocyte plays a role in transducing mechanical signals to biochemical signals that regulate bone remodeling. Membrane proteins such as S1P and AC6 are highly expressed in cilia and along cell processes, which act as mediators of osteocytes mechanotransduction and they may be suitable targets for development of therapeutic drugs treating bone metabolism disorder. S1P is required for the survival of osteoblasts and will facilitate bone formation. More S1Ps will be available by inhibiting the degradation of S1P, leading to improved bone health. Weske et al. showed better bone microarchitecture in their animal model after using the drug targeting S1P lyase [79]. This drug patent was filed and clinical testing was under preparation. AC6 can mediate the change in expression of bone remodeling genes. By increasing amount of AC6, bone formation can be enhanced and AC6 is a potential target in drug development.

Apart from signaling molecules released at later stage of mechanotransduction process, cytoskeleton fibres also play a role in relaying signals and have potential for further investigations. Cytoskeletons are responsible for maintenance of cell structure and normal function such as cell division and migration. Defective fibre proteins in bone cells will affect cell survival and can lead to impaired bone turnover. Bisphosphonates, as an example, are widely used for treatment of osteoporosis which can affect osteoclastic function by disrupting cytoskeleton [80]. Mulcahy et al. reported that bisphosphonates were used to treat *in vitro* models of osteocyte microinjury and bone remodeling. The shearing of osteocyte processes, with the presence of bisphosphonates, would modulate RANKL and OPG activities [81]. It is possible to further investigate the treatments of osteoporosis by regulating cytoskeleton activities such as opening of gated channels.

Osteoporotic patients with imbalanced bone turnover of low bone formation and high bone resorption during mechanical load-induced bone adaption may have problems in mechanosensing or mediating transduction of signals. As osteocyte morphology will deteriorate with age and diseases [82], therapies aiming at improving the signal transmission in mechanotransduction pathways can increase osteocytes’ responsiveness to mechanical stimulation. Zhou et al. showed that chlorogenic acid could prevent osteoporosis by interfering the PI3K/Akt pathway in their rat model, leading to increased osteoblast proliferation and bone formation [83]. Inhibition or augmentation of the targeted signaling molecules can regulate the availability of these molecules which restore the corresponding responses to mechanical load in diseased people. Ion channel proteins, β-catenin and sclerostin are some examples that can be developed for clinical use which are able to target mechanotransduction at different stages [[84], [85], [86]]. Romosozumab, for example, is a sclerostin inhibitor recently approved for treatment of post-menopausal osteoporosis and this drug can promote bone formation and inhibit bone resorption [87]. There is a need for developing new therapeutic drugs that can target different malfunctions in osteoporotic population.

As mechanical loading is required for the maintenance of bone metabolism, vibration treatment may be a potential intervention of imbalanced bone remodeling, which increases the amount of stimulated proteins and accelerate the bone metabolism in delayed bone growth. Osteocytes in osteoporotic bone are less sensitive and whole-body vibration treatment provides a higher chance of triggering signal transduction. ElDeeb et al. reported that the bone mineral density of post-menopausal women with osteoporosis had been improved after vibration treatment [88]. Choy et al. also demonstrated that vibration treatment could enhance osteocytes’ morphology and functions in rat osteoporotic bones [82]. This additional non-invasive mechanical stimulation is effective in restoring normal bone metabolism and becoming more common as a treatment.

There are few limitations in this review. The inclusion criteria of publications within 10 years may limit the scope of information on mechanotransduction especially the role of osteocyte in mechanobiology at tissue level. Studies that only use *in vitro* models cannot simulate the 3D structure of LCN and the signal transmission towards different directions and depth of bone matrix. Furthermore, *in vitro* studies only include single cell type but there are combinations of different cell types *in vivo* that coordinate with each other in harmony. Also, meta-analysis is not feasible due to the heterogeneity of the included studies.

In conclusion, this review has laid out current knowledge of factors and pathways involved in mechanotransduction that are specific to osteocytes for the regulation of bone metabolism. It is suggested that osteocytes’ unique morphology and its extensive network are involved in regulation of mechanotransduction. Mechanosensing proteins localised at gap junctions and cilia are the main mediators of upstream mechanotransduction pathway. Intermediate signaling proteins expressed at later stage of mechanotransduction play a mediating role and their changes in expression levels can induce activation of signal transduction pathways. Molecules regulating the activities of osteoblasts and osteoclasts can be targeted for further investigation towards the development of new therapeutic interventions that can treat people with bone adaptation disorder. These therapeutic targets will be suitable for population with impaired mechanosensitivity or prolonged bedrest.

## Author contributions

MCM Li: Data Curation, Writing - Original Draft; SKH Chow: Writing - Review & Editing; RMY Wong: Writing - Review & Editing; WH Cheung: Writing - Review & Editing, Supervision.

## Declaration of competing interest

The authors have no conflict of interest relevant to this review.

## References

[1] Hemmatian H., Bakker A.D., Klein-Nulend J., van Lenthe G.H. (2017). Aging, osteocytes, and mechanotransduction. Curr Osteoporos Rep.

[2] Turner C.H. (1998). Three rules for bone adaptation to mechanical stimuli. Bone.

[3] Spyropoulou A., Karamesinis K., Basdra E.K. (2015). Mechanotransduction pathways in bone pathobiology. Biochim Biophys Acta.

[4] Joiner D.M., Tayim R.J., Kadado A., Goldstein S.A. (2012). Bone marrow stromal cells from aged male rats have delayed mineralization and reduced response to mechanical stimulation through nitric oxide and ERK1/2 signaling during osteogenic differentiation. Biogerontology.

[5] Aguirre J.I., Plotkin L.I., Gortazar A.R., Millan M.M., O'Brien C.A., Manolagas S.C. (2007). A novel ligand-independent function of the estrogen receptor is essential for osteocyte and osteoblast mechanotransduction. J Biol Chem.

[6] Manolagas S.C., O'Brien C.A., Almeida M. (2013). The role of estrogen and androgen receptors in bone health and disease. Nat Rev Endocrinol.

[7] Kollmannsberger P., Kerschnitzki M., Repp F., Wagermaier W., Weinkamer R., Fratzl P. (2017). The small world of osteocytes: connectomics of the lacuno-canalicular network in bone. New J Phys.

[8] Klein-Nulend J., Bacabac R.G., Bakker A.D. (2012). Mechanical loading and how it affects bone cells: the role of the osteocyte cytoskeleton in maintaining our skeleton. Eur Cell Mater.

[9] Cowin S.C., Cardoso L. (2015). Blood and interstitial flow in the hierarchical pore space architecture of bone tissue. J Biomech.

[10] Weinbaum S., Cowin S.C., Zeng Y. (1994). A model for the excitation of osteocytes by mechanical loading-induced bone fluid shear stresses. J Biomech.

[11] Zhang C., Xu S., Zhang S., Liu M., Du H., Sun R. (2019). Ageing characteristics of bone indicated by transcriptomic and exosomal proteomic analysis of cortical bone cells. J Orthop Surg Res.

[12] Santos A., Bakker A.D., Klein-Nulend J. (2009). The role of osteocytes in bone mechanotransduction. Osteoporos Int.

[13] Joiner D.M., Tayim R.J., McElderry J.D., Morris M.D., Goldstein S.A. (2014). Aged male rats regenerate cortical bone with reduced osteocyte density and reduced secretion of nitric oxide after mechanical stimulation. Calcif Tissue Int.

[14] Kennedy O.D., Herman B.C., Laudier D.M., Majeska R.J., Sun H.B., Schaffler M.B. (2012). Activation of resorption in fatigue-loaded bone involves both apoptosis and active pro-osteoclastogenic signaling by distinct osteocyte populations. Bone.

[15] Wang L., You X., Lotinun S., Zhang L., Wu N., Zou W. (2020). Mechanical sensing protein PIEZO1 regulates bone homeostasis via osteoblast-osteoclast crosstalk. Nat Commun.

[16] Lanyon L.E., Rubin, Raisz, Marotti Lees (1993). Osteocytes, strain detection, bone modeling and remodeling. Calcif Tissue Int.

[17] Zhou T., Gao B., Fan Y., Liu Y., Feng S., Cong Q. (2020). Piezo1/2 mediate mechanotransduction essential for bone formation through concerted activation of NFAT-YAP1-ss-catenin. Elife.

[18] Sun W., Chi S., Li Y., Ling S., Tan Y., Xu Y. (2019). The mechanosensitive Piezo1 channel is required for bone formation. Elife.

[19] Tatsumi S., Ishii K., Amizuka N., Li M., Kobayashi T., Kohno K. (2007). Targeted ablation of osteocytes induces osteoporosis with defective mechanotransduction. Cell Metabol.

[20] Burra S., Nicolella D.P., Francis W.L., Freitas C.J., Mueschke N.J., Poole K. (2010). Dendritic processes of osteocytes are mechanotransducers that induce the opening of hemichannels. Proceed. Natl. Acad. Sci. U.S.A.

[21] de Castro L.F., Maycas M., Bravo B., Esbrit P., Gortazar A. (2015). VEGF receptor 2 (VEGFR2) activation is essential for osteocyte survival induced by mechanotransduction. J Cell Physiol.

[22] Dobrosak C., Gooi J.H. (2017). Increased sphingosine-1-phosphate production in response to osteocyte mechanotransduction. Bone Rep.

[23] Gortazar A.R., Martin-Millan M., Bravo B., Plotkin L.I., Bellido T. (2013). Crosstalk between caveolin-1/extracellular signal-regulated kinase (ERK) and beta-catenin survival pathways in osteocyte mechanotransduction. J Biol Chem.

[24] Haugh M.G., Vaughan T.J., McNamara L.M. (2015). The role of integrin alpha(V)beta(3) in osteocyte mechanotransduction. J Mech Behav Biomed Mater.

[25] Hum J.M., Day R.N., Bidwell J.P., Wang Y., Pavalko F.M. (2014). Mechanical loading in osteocytes induces formation of a Src/Pyk2/MBD2 complex that suppresses anabolic gene expression. PloS One.

[26] Kringelbach T.M., Aslan D., Novak I., Ellegaard M., Syberg S., Andersen C.K. (2015). Fine-tuned ATP signals are acute mediators in osteocyte mechanotransduction. Cell Signal.

[27] Kwon R.Y., Temiyasathit S., Tummala P., Quah C.C., Jacobs C.R. (2010). Primary cilium-dependent mechanosensing is mediated by adenylyl cyclase 6 and cyclic AMP in bone cells. Faseb J : Off Publ Feder Am Soc Exp Biol.

[28] Lee K.L., Guevarra M.D., Nguyen A.M., Chua M.C., Wang Y., Jacobs C.R. (2015). The primary cilium functions as a mechanical and calcium signaling nexus. Cilia.

[29] Li X., Liu C., Li P., Li S., Zhao Z., Chen Y. (2013). Connexin 43 is a potential regulator in fluid shear stress-induced signal transduction in osteocytes. J Orthop Res.

[30] Lyons J.S., Joca H.C., Law R.A., Williams K.M., Kerr J.P., Shi G. (2017). Microtubules tune mechanotransduction through NOX2 and TRPV4 to decrease sclerostin abundance in osteocytes. Sci Signal.

[31] Moore E.R., Ryu H.S., Zhu Y.X., Jacobs C.R. (2018). Adenylyl cyclases and TRPV4 mediate Ca(2+)/cAMP dynamics to enhance fluid flow-induced osteogenesis in osteocytes. J Mol Biochem.

[32] Ren J., Wang X.H., Wang G.C., Wu J.H. (2013). 17beta estradiol regulation of connexin 43-based gap junction and mechanosensitivity through classical estrogen receptor pathway in osteocyte-like MLO-Y4 cells. Bone.

[33] Santos A., Bakker A.D., Zandieh-Doulabi B., de Blieck-Hogervorst J.M., Klein-Nulend J. (2010). Early activation of the beta-catenin pathway in osteocytes is mediated by nitric oxide, phosphatidyl inositol-3 kinase/Akt, and focal adhesion kinase. Biochem Biophys Res Commun.

[34] Sasaki F., Hayashi M., Mouri Y., Nakamura S., Adachi T., Nakashima T. (2020). Mechanotransduction via the Piezo1-Akt pathway underlies Sost suppression in osteocytes. Biochem Biophys Res Commun.

[35] Wu X.T., Sun L.W., Yang X., Ding D., Han D., Fan Y.B. (2017). The potential role of spectrin network in the mechanotransduction of MLO-Y4 osteocytes. Sci Rep.

[36] Zhang J.N., Zhao Y., Liu C., Han E.S., Yu X., Lidington D. (2015). The role of the sphingosine-1-phosphate signaling pathway in osteocyte mechanotransduction. Bone.

[37] Bivi N., Pacheco-Costa R., Brun L.R., Murphy T.R., Farlow N.R., Robling A.G. (2013). Absence of Cx43 selectively from osteocytes enhances responsiveness to mechanical force in mice. J Orthop Res.

[38] Corry K.A., Zhou H., Brustovetsky T., Himes E.R., Bivi N., Horn M.R. (2019). Stat3 in osteocytes mediates osteogenic response to loading. Bone Rep.

[39] Lee K.L., Hoey D.A., Spasic M., Tang T., Hammond H.K., Jacobs C.R. (2014). Adenylyl cyclase 6 mediates loading-induced bone adaptation in vivo. Faseb J : Off Publ Feder Am Soc Exp Biol.

[40] Morrell A.E., Brown G.N., Robinson S.T., Sattler R.L., Baik A.D., Zhen G. (2018). Mechanically induced Ca(2+) oscillations in osteocytes release extracellular vesicles and enhance bone formation. Bone Res.

[41] Hagan M.L., Yu K., Zhu J., Vinson B.N., Roberts R.L., Montesinos Cartagena M. (2020). Decreased pericellular matrix production and selection for enhanced cell membrane repair may impair osteocyte responses to mechanical loading in the aging skeleton. Aging Cell.

[42] Cabahug-Zuckerman P., Stout R.F., Majeska R.J., Thi M.M., Spray D.C., Weinbaum S. (2018). Potential role for a specialized beta3 integrin-based structure on osteocyte processes in bone mechanosensation. J Orthop Res.

[43] Hu M., Tian G.W., Gibbons D.E., Jiao J., Qin Y.X. (2015). Dynamic fluid flow induced mechanobiological modulation of in situ osteocyte calcium oscillations. Arch Biochem Biophys.

[44] Jing D., Baik A.D., Lu X.L., Zhou B., Lai X., Wang L. (2014). In situ intracellular calcium oscillations in osteocytes in intact mouse long bones under dynamic mechanical loading. Faseb J : Off Publ Feder Am Soc Exp Biol.

[45] Kalogeropoulos M., Varanasi S.S., Olstad O.K., Sanderson P., Gautvik V.T., Reppe S. (2010). Zic1 transcription factor in bone: neural developmental protein regulates mechanotransduction in osteocytes. Faseb J : Off Publ Feder Am Soc Exp Biol.

[46] Liu C., Zhao Y., Cheung W.Y., Gandhi R., Wang L., You L. (2010). Effects of cyclic hydraulic pressure on osteocytes. Bone.

[47] Li X., Han L., Nookaew I., Mannen E., Silva M.J., Almeida M. (2019). Stimulation of Piezo1 by mechanical signals promotes bone anabolism. Elife.

[48] Meshcheryakova A., Mechtcheriakova D., Pietschmann P. (2017). Sphingosine 1-phosphate signaling in bone remodeling: multifaceted roles and therapeutic potential. Expert Opin Ther Targets.

[49] Koyabu Y., Nakata K., Mizugishi K., Aruga J., Mikoshiba K. (2001). Physical and functional interactions between Zic and Gli proteins. J Biol Chem.

[50] You L.D., Cowin S.C., Schaffler M.B., Weinbaum S. (2001). A model for strain amplification in the actin cytoskeleton of osteocytes due to fluid drag on pericellular matrix. J Biomech.

[51] Han Y., Cowin S.C., Schaffler M.B., Weinbaum S. (2004). Mechanotransduction and strain amplification in osteocyte cell processes. Proc Natl Acad Sci U S A.

[52] Fletcher D.A., Mullins D. (2010). Cell mechanics and the cytoskeleton. Nature.

[53] Wang Y., McNamara L.M., Schaffler M.B., Weinbaum S. (2008). Strain amplification and integrin based signaling in osteocytes. J Musculoskelet Neuronal Interact.

[54] Sun Z.Q., Guo S.S., Fassler R. (2016). Integrin-mediated mechanotransduction. J Cell Biol.

[55] Jaqaman K., Grinstein S. (2012). Regulation from within: the cytoskeleton in transmembrane signaling. Trends Cell Biol.

[56] Dallas S.L., Prideaux M., Bonewald L.F. (2013). The osteocyte: an endocrine cell . . . and more. Endocr Rev.

[57] Plotkin L.I., Speacht T.L., Donahue H.J. (2015). Cx43 and mechanotransduction in bone. Curr Osteoporos Rep.

[58] Middleton K., Kondiboyina A., Borrett M., Cui Y., Mei X., You L. (2018). Microfluidics approach to investigate the role of dynamic similitude in osteocyte mechanobiology. J Orthop Res.

[59] Xu L.C.H., Shao H., Ma Y.H.V., You L.D. (2019). OCY454 osteocytes as an in vitro cell model for bone remodeling under mechanical loading. J Orthop Res.

[60] Lewis K.J., Frikha-Benayed D., Louie J., Stephen S., Spray D.C., Thi M.M. (2017). Osteocyte calcium signals encode strain magnitude and loading frequency in vivo. Proceed Natl Acad Sci USA.

[61] Thouverey C., Caverzasio J. (2015). Sclerostin inhibits osteoblast differentiation without affecting BMP2/SMAD1/5 or Wnt3a/beta-catenin signaling but through activation of platelet-derived growth factor receptor signaling in vitro. BoneKEy Rep.

[62] Song F.L., Jiang D.W., Wang T.C., Wang Y., Lou Y., Zhang Y.Q. (2017). Mechanical stress regulates osteogenesis and adipogenesis of rat mesenchymal stem cells through PI3K/Akt/GSK-3 beta/beta-Catenin signaling pathway. BioMed Res Int.

[63] Tan S.D., Bakker A.D., Semeins C.M., Kuijpers-Jagtman A.M., Klein-Nulend J. (2008). Inhibition of osteocyte apoptosis by fluid flow is mediated by nitric oxide. Biochem Biophys Res Commun.

[64] Chen J.Q., Long F.X. (2013). Beta-catenin promotes bone formation and suppresses bone resorption in postnatal growing mice. J Bone Miner Res.

[65] Lara-Castillo N., Kim-Weroha N.A., Kamel M.A., Javaheri B., Ellies D.L., Krumlauf R.E. (2015). In vivo mechanical loading rapidly activates beta-catenin signaling in osteocytes through a prostaglandin mediated mechanism. Bone.

[66] Burgers T.A., Williams B.O. (2013). Regulation of Wnt/beta-catenin signaling within and from osteocytes. Bone.

[67] Xiong J., O'Brien C.A. (2012). Osteocyte RANKL: new insights into the control of bone remodeling. J Bone Miner Res : off J Am Soc Bone Miner Res.

[68] Hwang S.Y., Putney J.W. (2011). Calcium signaling in osteoclasts. Bba-Mol Cell Res.

[69] Lau E., Al-Dujaili S., Guenther A., Liu D., Wang L., You L. (2010). Effect of low-magnitude, high-frequency vibration on osteocytes in the regulation of osteoclasts. Bone.

[70] Li J., Rose E., Frances D., Sun Y., You L. (2012). Effect of oscillating fluid flow stimulation on osteocyte mRNA expression. J Biomech.

[71] Willoughby D., Cooper D.M.F. (2007). Organization and Ca2+ regulation of adenylyl cyclases in cAMP microdomains. Physiol Rev.

[72] Kitase Y., Barragan L., Qing H., Kondoh S., Jiang J.X., Johnson M.L. (2010). Mechanical induction of PGE(2) in osteocytes blocks glucocorticoid-induced apoptosis through both the beta-catenin and PKA pathways. J Bone Miner Res.

[73] Kaji H., Sugimoto T., Kanatani M., Fukase M., Kumegawa M., Chihara K. (1996). Prostaglandin E(2) stimulates osteoclast-like cell formation and bone-resorbing activity via osteoblasts: role of cAMP-dependent protein kinase. J Bone Miner Res.

[74] Amano S., Naganuma K., Kawata Y., Kawakami K., Kitano S., Hanazawa S. (1996). Prostaglandin E(2) stimulates osteoclast formation via endogenous IL-1 beta expressed through protein kinase A. J Immunol.

[75] Buenzli P.R., Sims N.A. (2015). Quantifying the osteocyte network in the human skeleton. Bone.

[76] Wang L.Y. (2018). Solute transport in the bone lacunar-canalicular system (LCS). Curr Osteoporos Rep.

[77] Cowin S.C., Weinbaum S., Zeng Y. (1995). A case for bone canaliculi as the anatomical site of strain generated potentials. J Biomech.

[78] Corrigan M.A., Johnson G.P., Stavenschi E., Riffault M., Labour M.N., Hoey D.A. (2018). TRPV4-mediates oscillatory fluid shear mechanotransduction in mesenchymal stem cells in part via the primary cilium. Sci Rep.

[79] Weske S., Vaidya M., Reese A., von Wnuck Lipinski K., Keul P., Bayer J.K. (2018). Targeting sphingosine-1-phosphate lyase as an anabolic therapy for bone loss. Nat Med.

[80] Santora A.C., Sharma A., Leder B.Z., Wein M.N. (2020). Bisphosphonates: Mechanisms of Action and Role in Osteoporosis Therapy. Osteoporosis: Pathophysiology and Clinical Management.

[81] Mulcahy L.E., Curtin C.M., McCoy R.J., O'Brien F.J., Taylor D., Lee T.C. (2015). The effect of bisphosphonate treatment on the biochemical and cellular events during bone remodelling in response to microinjury stimulation. Eur Cell Mater.

[82] Choy M.V., Wong R.M., Li M.C., Wang B.Y., Liu X.D., Lee W. (2020). Can we enhance osteoporotic metaphyseal fracture healing through enhancing ultrastructural and functional changes of osteocytes in cortical bone with low-magnitude high-frequency vibration?. Faseb J : Off Publ Feder Am Soc Exp Biol.

[83] Zhou R.P., Lin S.J., Wan W.B., Zuo H.L., Yao F.F., Ruan H.B. (2016). Chlorogenic acid prevents osteoporosis by Shp2/PI3K/Akt pathway in ovariectomized rats. PloS One.

[84] Haustrate A., Hantute-Ghesquier A., Prevarskaya N., Lehen'kyi V. (2019). Monoclonal antibodies targeting ion channels and their therapeutic potential. Front Pharmacol.

[85] Kim J.H., Liu X., Wang J., Chen X., Zhang H., Kim S.H. (2013). Wnt signaling in bone formation and its therapeutic potential for bone diseases. Ther Adv Musculoskelet Dis.

[86] Recker R.R., Benson C.T., Matsumoto T., Bolognese M.A., Robins D.A., Alam J. (2015). A randomized, double-blind phase 2 clinical trial of blosozumab, a sclerostin antibody, in postmenopausal women with low bone mineral density. J Bone Miner Res : off J Am Soc Bone Miner Res.

[87] Shakeri A., Adanty C. (2020). Romosozumab (sclerostin monoclonal antibody) for the treatment of osteoporosis in postmenopausal women: a review. J Popul Ther Clin Pharmacol.

[88] Abeer M., ElDeeb A.A.A.-A. (2020). Effect of whole-body vibration exercise on power profile and bone mineral density in postmenopausal women with osteoporosis: a randomized controlled trial. J Manipulative Physiol Therapeut.

